# Outcomes and surgical technique of laparoscopic hysterectomy using an articulating laparoscopic instrument: a prospective observational study

**DOI:** 10.3389/fsurg.2025.1491767

**Published:** 2025-06-24

**Authors:** Jun-Hyeong Seo, Won-Ji Kim, Chel Hun Choi, Tae-Joong Kim, Jeong-Won Lee, Byeong-Gie Kim, Kazuyoshi Kato, Yi-Liang Lee, Yu-Li Chen, Yoo-Young Lee

**Affiliations:** ^1^ Gynecologic Cancer Center, Department of Obstetrics and Gynecology, Samsung Medical Center, Sungkyunkwan University School of Medicine, Seoul, Republic of Korea; ^2^ Department of Obstetrics and Gynecology, Kitasato University School of Medicine, Sagamihara, Japan; ^3^ Department of Obstetrics and Gynecology, Tri-Service General Hospital National Defense Medical Center, Taipei, Taiwan; ^4^ Department of Obstetrics and Gynecology, College of Medicine, National Taiwan University, Taipei, Taiwan

**Keywords:** articulating instrument, laparoscopic hysterectomy, multi-joint mechanism, surgical skill, surgical video

## Abstract

**Objective:**

This study aimed to evaluate the feasibility of performing laparoscopic hysterectomy using an articulating laparoscopic instrument that provides flexible and ergonomic movements similar to robotic systems and to demonstrate the surgical techniques facilitated by this advanced instrument.

**Methods:**

This prospective observational study was performed at a single institution. We reviewed the electronic medical records of patients who underwent laparoscopic hysterectomy using an articulating laparoscopic instrument between October 2022 and May 2023. The surgeries were performed by two surgeons, both experienced in laparoscopic hysterectomy. Data on patient demographics, clinical outcomes, as well as pathological and postoperative results, were collected prospectively for analysis.

**Results:**

Of the 100 patients enrolled in this prospective observational study utilizing the articulating laparoscopic instrument for gynecologic surgery, 45 underwent hysterectomy with this instrument. All procedures were successfully completed laparoscopically without the need for conversion to laparotomy. Indications for hysterectomy were benign in 55.5% of the patients, borderline in 6.6%, and malignant in 37.7%. Median operative time was 78 min (range, 44–156 min). The median uterine weight was 203.5 g (range, 43–875 g), and the median estimated blood loss was 100 ml (range, 50–300 ml). The median length of hospital stay postoperatively was 2 days (range, 2–4 days). Postoperative complications were observed in six (13.3%) patients, with one developing a vesicovaginal fistula as a delayed complication.

**Conclusion:**

The results of this study present the practicality of employing the articulating laparoscopic instrument in laparoscopic hysterectomy, highlighting its efficacy in improving surgical technique. The enhanced maneuverability provided by this instrument allows for precise and efficient operations, demonstrating its value in performing complex surgical procedures.

## 1 Introduction

Hysterectomy is one of the most frequently performed surgical procedures in gynecology, with a prevalence of 21.1% among women in the United States as of 2016 (1). The choice of approach to hysterectomy, whether abdominal, laparoscopic, or vaginal, depends on various factors, including patient characteristics, the type of underlying disease, and the surgeon's preference. Since its introduction in 1989, total laparoscopic hysterectomy (TLH) has become the preferred standard for the management of benign uterine conditions (2). Total laparoscopic hysterectomy offers several advantages over abdominal hysterectomy, such as reduced blood loss, shorter hospital stays, and a lower complication rate, which collectively contribute to faster patient recovery (3). Moreover, advancements in surgical techniques and technology have facilitated the broader adoption of TLH in the early stages of gynecologic oncology. When comparing conventional laparotomy with laparoscopic surgery for early-stage gynecologic cancers, studies have demonstrated equivalent survival outcomes, with the added benefits of lower perioperative morbidity, enhanced quality of life, shorter hospital stays, and faster postoperative recovery, particularly in endometrial cancer treatment (4). However, despite these advantages, laparoscopic surgery is not without its challenges. The confined working space inherent to laparoscopy imposes restrictions on instrument movement, which can diminish surgical dexterity and limit the precision with which tissues are manipulated (5, 6).

In contrast to conventional laparoscopy, robotic surgery incorporates a multi-joint mechanism, three-dimensional (3D) vision, and enhanced ergonomics. These features help to overcome the limitations of traditional laparoscopic surgery and provide significant advantages in terms of surgical dexterity and precision. Studies have shown that robotic surgery is associated with shorter hospital stays and reduced need for blood transfusions compared to open surgery, and it is comparable to laparoscopic surgery regarding complication rates and conversions to laparotomy (7). However, the cost-effectiveness of robotic surgery remains questionable when compared to conventional laparoscopy (8). Several studies have explored the use of these articulating laparoscopic instruments across various surgical specialties, including gastrectomy, low anterior resection, appendectomy, and pediatric thoracoscopic thymectomy, to assess their efficacy in enhancing laparoscopic procedures (9–13). Despite these advances, there has been limited study evaluating the use of articulating laparoscopic instruments in gynecologic surgery. Therefore, the objective of this prospective observational study is to demonstrate the feasibility of laparoscopic hysterectomy performed with a multi-joint articulating instrument and to illustrate the specific surgical techniques enabled by this advanced technology.

## 2 Materials and methods

### 2.1 Study population and general information

This prospective observational study was conducted at a single institution. Among patients enrolled in a study utilizing an articulating laparoscopic instrument in gynecologic surgery, we analyzed the electronic medical records of those who underwent laparoscopic hysterectomy between October 2022 and May 2023. Adult women (≥19 years of age) scheduled for laparoscopic hysterectomy were eligible for inclusion. Exclusion criteria included current pregnancy and inability to comprehend the study. Written informed consent was obtained from all the participants for the use of the articulating laparoscopic instrument, de-identification of collected data, scientific analysis, and publication of findings. The Institutional Review Board (IRB) of Samsung Medical Center approved this study (IRB No. 2022-08-151).

### 2.2 Surgical procedures

All surgical procedures were performed by two high-volume surgeons (Chel Hun Choi and Yoo-Young Lee) with >10 years of experience, each performing >50 hysterectomies annually (14). In the operating room, patients were positioned in the low lithotomy Trendelenburg position. The lead surgeon stood on the patient's left side, with the first assistant positioned on the right side holding the camera, and an additional assistant for uterine manipulation positioned between the patient's legs.

A commercial four-lumen trocar system (Gloveport®; Nelis, Bucheon, South Korea) was initially inserted through a transumbilical incision using the Hasson's technique (15). Following insufflation with carbon dioxide to a pressure of 11 mmHg, a laparoscope was introduced through one channel of the umbilical port, with an articulating instrument inserted via another channel. To enhance surgical traction and maneuverability, a 5-mm suprapubic trocar was routinely placed for the use of a grasper. Depending on the complexity of the procedure and the surgeon's preference, additional trocars (either 5- or 12-mm) were selectively inserted in the lower abdominal quadrants. As such, the majority of procedures employed a dual-port or multi-port approach rather than a strictly single-port approach.

**Figure 1:**
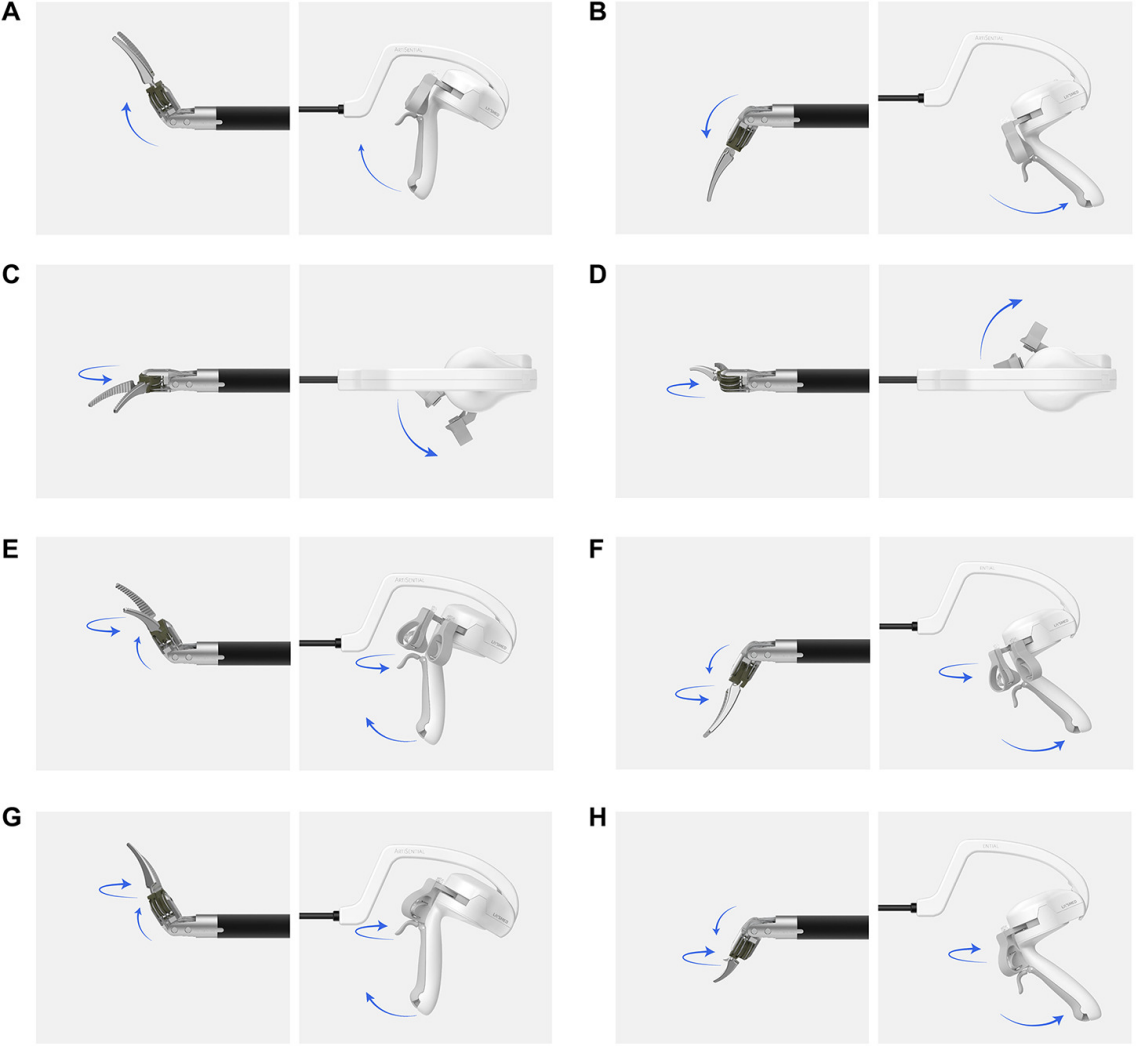
Movement of the end-effector part of an articulating device in response to the handle grip. **(A)** Up, **(B)** down, **(C)** left, **(D)** right, **(E)** up-left, **(F)** down-left, **(G)** up-right, **(H)** down-right. The image illustrates how the end-effector's multi-directional movement is directly controlled by the surgeon's wrist and hand through the instrument's handle. This mechanism mimics the articulation seen in robotic surgery, allowing precise maneuvering in confined spaces.

To ensure ergonomic positioning and optimal visualization, the primary monitor was placed at the patient's foot side, to the right of the assistant. A secondary monitor was routinely used and positioned at the patient's head side, to the right of the operator, allowing the assistant to maintain a comfortable and efficient viewing angle. This dual-monitor setup enabled coordinated teamwork and minimized physical strain for both surgeons and assistants. The ArtiSential® (LIVSMED Inc., South Korea) articulating laparoscopic instrument, which offers multi-degree-of-freedom movement akin to a robotic system, was employed (16). The overall appearance of the articulating instrument and movement of the end effector, according to the handle grip movement of the articulating laparoscopic instrument, is shown in Figure 1. During hysterectomy, tissue dissection—particularly during colpotomy—was performed using monopolar energy delivered via either articulating instruments or conventional laparoscopic instruments, depending on surgeon preference and intraoperative considerations. For vascular pedicles such as the uterine artery and infundibulopelvic ligament, bipolar energy was routinely applied via conventional instruments to ensure secure hemostasis. Additionally, advanced energy devices were selectively utilized depending on procedural complexity and surgeon discretion.

The vaginal cuff was closed laparoscopically using continuous 2-0 absorbable barbed sutures, applied with either conventional straight Maryland needle holders or articulating needle holders, according to the surgeon's preference. When performing a hysterectomy using the articulating instrument, three key steps are essential:

#### 2.2.1 Creation of avascular spaces

The broad ligament is opened to create pararectal and paravesical spaces, which allow for visualization of the ureter, uterine artery, and hypogastric artery, thereby reducing the risk of injury. When creating the vesicocervical space, articulating instruments allow for smooth dissection, similar to robotic surgery, gently pushing the bladder away, and minimizing the risk of bladder injury. Articulating instruments are utilized to precisely dissect these spaces without necessitating extensive arm movement. Supplementary Video S1 demonstrates the creation of pararectal, paravesical, and vesicocervical spaces using articulating instruments.

#### 2.2.2 Colpotomy

Following resection of the uterosacral and cardinal ligaments, colpotomy is performed. This step is particularly challenging in conventional multiport and dual-port surgery due to the deep pelvic location, often inaccessible to straight instruments. Articulating instruments overcome this challenge by allowing the end effector to maneuver in any direction, even in restricted spaces, as demonstrated in Supplementary Video S2.

#### 2.2.3 Vaginal cuff closure

After colpotomy, the vaginal cuff is sutured laparoscopically. In dual-port surgery, the near-perpendicular angle between the vaginal cuff and the laparoscopic needle holder can complicate this procedure. Articulating needle holders allow surgeons to adjust the suture direction using wrist movements, similar to a robotic system, ensuring precise suture placement even in challenging angles (Supplementary Video S3).

### 2.3 Data collection

Data were prospectively collected (http://mavencdms.com) on patient characteristics, including age at operation, preoperative body mass index (BMI), number of prior abdominal surgeries, preoperative anemia status, and American Society of Anesthesiologists (ASA) score. Clinical outcomes included the number of laparoscopic ports used, types of articulating instruments employed, total operative time (from skin incision to final suture), time to vaginal stump closure (from first to last suture), uterine weight (measured postoperatively), estimated blood loss (EBL; based on suction device contents), transfusion requirement, and conversion to laparotomy rate. Pathologic outcomes were assessed based on preoperative tumor types, tumor location, and final postoperative diagnosis. Postoperative outcomes included the length of hospital stay, reduction in hemoglobin (difference between preoperative and first postoperative day hemoglobin), and postoperative complications and morbidity.

### 2.4 Statistical analysis

Statistical analyses were performed using SPSS software version 28.0 (IBM SPSS Statistics®, Armonk, NY, USA). All the analyses were conducted using available data. Categorical variables were analyzed using the Chi-square test or Fisher's exact test, while continuous variables were assessed using the Student's *t*-test or Mann–Whitney *U* test. Mean values are presented with standard errors (±SE), and median values are provided as ranges. Statistical significance was set at *p* < 0.05.

## 3 Results

A total of 100 patients were enrolled in this prospective observational study using an articulating laparoscopic instrument in gynecologic surgery. Of these, 45 patients underwent hysterectomy and were included in this analysis. The demographic characteristics of the patients are summarized in Table 1. The mean age of the patients at the time of surgery was 47.6 ± 8.6 years, and the mean BMI was 24.9 ± 5.1 kg/m^2^. Eighteen patients (40%) had a history of >1 prior abdominal surgery. Ten patients (22.2%) had preoperative anemia, defined as a hemoglobin level of <11 g/dl. The majority of the patients (97.7%) had an ASA score of I or II at the time of surgery.

**Table 1:** Patient demographics.

Variables	Values
Age at operation (years)	47.6 ± 8.6
BMI (kg/m^2^)	24.9 ± 5.1
Number of prior abdominal surgeries	
0	27 (60.0)
1	16 (35.5)
≥2	2 (4.4)
Preoperative anemia (Hb < 11 g/dl)	10 (22.2)
ASA score	
I	4 (8.8)
II	40 (88.9)
III/IV^a^	1 (2.2)

Data are presented as mean ± SD or No. (%). BMI, body mass index; Hb, hemoglobin; ASA, American Society of Anesthesiologists; SD, standard deviation.

^a^ ASA score III and IV were collapsed into a single category because only 1 patient had score III.

### 3.1 Clinical outcomes

The clinical outcomes of patients undergoing surgery with the articulating laparoscopic instrument are summarized in Table 2. The most common port configuration was dual-port access (64.4%), typically involving an umbilical and a suprapubic port. In nearly all cases, a single type of articulating instrument was employed, with monopolar scissors being the most frequently utilized (53.3%), followed by needle holders (33.3%) and monopolar hooks (15.5%).

**Table 2:** Clinical outcomes.

Variables	Values
Number of ports	
2	29 (64.4)
3+	16 (35.5)
Used an articulating instrument	
Monopolar scissors	24 (53.3)
Monopolar hook	7 (15.5)
Needle holder	15 (33.3)
Total operative time (min)	78 (44–156)
Time to vaginal stump closure (min)	
Using articulating needle holder	11 (7–14)
Using straight needle holder	4 (2–17)
Weight of uterus (g)	203.5 (43–875)
EBL (ml)	100 (50–300)
Transfusion	1 (2.2)
Conversion rate	0 (0.0)

Data are presented as median (range) or No. (%). EBL, estimated blood loss.

The median total operation time was 78 min (range, 44–156 min). Vaginal stump closure was performed using articulating needle holders in 15 patients (33.3%), with a median suturing time of 11 min (range, 7–14 min). In contrast, when conventional straight needle holders were used, the median closure time was shorter, at 4 min (range, 2–17 min). The median weight of the uterus after surgery was 203.5 g (range, 43–875 g), and the estimated blood loss was 100 ml (range, 50–300 ml). Only one patient required intraoperative blood transfusion. All procedures were completed laparoscopically without conversion to laparotomy.

### 3.2 Pathologic outcomes

The pathologic outcomes are detailed in Table 3. Preoperative imaging suggested that the pathology was benign in 25 patients (55.5%), borderline in 3 patients (6.6%), and malignant in 17 patients (37.7%). The tumor was most commonly located in the uterus (80%). The final pathologic diagnoses included leiomyoma (33.3%), endometrial cancer (20.0%), cervical cancer (20.0%), and adenomyosis (15.5%). Additionally, three patients had endometrial hyperplasia, one had a high-grade squamous intraepithelial lesion, and one had ovarian cancer.

**Table 3:** Pathologic outcomes.

Variables	Values
Preoperative type of tumor	
Benign	25 (55.5)
Borderline	3 (6.6)
Malignant	17 (37.7)
Preoperative location of tumors	
Uterus	36 (80)
Adnexa	3 (6.6)
Cervix	6 (13.3)
Diagnosis of tumors after surgery	
Leiomyoma	15 (33.3)
Adenomyosis	7 (15.5)
Endometrial cancer	9 (20.0)
Cervical cancer	9 (20.0)
Others	5 (11.1)

Data are presented as No. (%).

### 3.3 Intraoperative and postoperative outcomes

Intraoperative and postoperative outcomes are presented in Table 4. The median length of hospital stay was 2 days (range, 2–4 days). The mean decrease in hemoglobin level on postoperative day 1 was 1.5 ± 0.8 g/dl. No intraoperative complications were reported. Six patients (13.3%) experienced at least one postoperative complication. Complications were categorized as early (within 30 days postoperatively) and late (after 30 days). Early complications included anemia (2.2%), postoperative vaginal bleeding (4.4%), and urinary tract or wound infections (4.4%). In one patient who developed anemia, the hemoglobin level decreased from 14.0 g/dl preoperatively to 7.9 g/dl on postoperative day 2. One patient developed a late complication, a vesicovaginal fistula. This was the only case classified as Clavien–Dindo classification (CDC) grade III or higher. Further details of this case will be discussed in the Discussion section.

**Table 4:** Intraoperative and postoperative outcomes.

Variables	Values
Hospital stays (days)	2 (2–4)
Hemoglobin decrease (g/dl)	1.5 ± 0.8
Intraoperative complication	0 (0.0)
Postoperative complication	6 (13.3)
Early complication	
Anemia	1 (2.2)
Vaginal bleeding	2 (4.4)
Infection	2 (4.4)
Late complication	
Vesicovaginal fistula	1 (2.2)
Morbidity (CDC grade III–IV)	1 (2.2)

Data are presented as mean ± SD, median (range), or No. (%). CDC, Clavien-Dindo classification; SD, standard deviation.

## 4 Discussion

In recent years, there has been a growing trend towards less invasive procedures in gynecological surgery, leading to the widespread adoption of minimally invasive surgery (MIS) for both benign and malignant gynecologic tumors. The technology associated with MIS has advanced significantly, and conventional multi-port laparoscopic surgery has been largely supplanted by reduced-port laparoscopic techniques (17). However, conventional laparoscopic surgery commonly relies on straight, fixed instruments, which are limited in range of motion and ergonomics, making surgical procedures more challenging for surgeons. At the same time, robot-assisted laparoscopy has gained popularity due to its 3D optics, enhanced range of motion, and superior ergonomics. Nevertheless, the high cost of robotic platforms limits their accessibility to a broader patient population (18). Consequently, multi-articulating laparoscopic instruments, which enhance the range of motion and improve ergonomics, are emerging as a more cost-effective alternative to address the limitations of conventional laparoscopy.

This study reports on the experience of TLH using articulating laparoscopic instruments at a single medical center, and is the first to demonstrate the surgical techniques of hysterectomy with articulating instruments through video documentation. The median BMI of the patients in our study was 24.9 ± 5.1 kg/m^2^, which is comparable to the mean BMI of 24.4 ± 4.6 kg/m^2^ reported in an earlier study among Asian populations (19). In a 2011 study, 100 cases of single-port TLH using conventional laparoscopic instruments reported a median operation time of 80 min and complication rate of 8% (20). Although direct comparison with the present study, which included dual-port and multi-port techniques, is difficult, the median operation time with articulating instruments was 78 min, and the complication rate was 13.3%. Previous studies have reported overall complication rates of 5.8%–11.5% and major complication rates of 2.2%–2.7% for laparoscopic hysterectomy in benign uterine disease (21). Similarly, our study found an overall complication rate of 13.3% and a CDC grade III or higher morbidity rate of 2.2%.

Among the six patients who experienced postoperative complications, one patient with stage IB2 cervical cancer developed a CDC grade III vesicovaginal fistula following laparoscopic radical hysterectomy. This patient had no history of previous abdominal surgery other than a cesarean section, and no adhesions were observed during the procedure. Articulating laparoscopic instruments were used for pelvic lymph node dissection and colpotomy, while conventional straight needle holders were used for vaginal stump closure. The patient presented with vaginal discharge 1 month postoperatively, and cystoscopy confirmed a vesicovaginal fistula. Although surgery was initially planned, the fistula resolved spontaneously, and no further complications have occurred. Previous studies have reported a 1% incidence of vesicovaginal fistula following radical hysterectomy, with a potentially higher risk associated with the laparoscopic approach (22). In our study, the incidence of this complication was 2.2%. However, given the small sample size and the fact that articulating instruments were not used during vaginal stump closure, this difference in incidence cannot be directly attributed to the use of articulating instruments.

Subgroup analysis revealed that the median time to perform vaginal stump closure using the articulating instrument was significantly longer compared to the time using straight needle holders: 11 min (range, 7–14 min) vs. 4 min (range, 2–17 min; *p* < 0.001). According to previous studies, a learning curve of at least 10–15 cases is required to achieve proficiency in laparoscopic hysterectomy (23, 24). The significant difference observed in our study may be due to the surgeons' position on this learning curve with the articulating instruments. However, since a defined learning curve for hysterectomy using articulating laparoscopic instruments has not yet been established, further studies are warranted. Additionally, a 2013 study found no significant difference in perioperative outcomes based on uterine weight in laparoscopic hysterectomy (25). We analyzed outcomes based on a uterine weight threshold of 500 g when using the articulating instrument and found no significant differences in perioperative outcomes between the two groups (Supplementary Table S1). This suggests that the articulating instrument can be effectively used for operating on larger uteri weighing >500 g.

The use of articulating instruments in real-world surgery presents several advantages and disadvantages. The ability to obtain various angles in the confined space of the pelvis without being restricted by spatial limitations allows for quick hemostasis and dissection from any angle using articulating instruments. When utilized through a single umbilical port, these instruments enable the surgeon to perform procedures in the desired position without interference from the camera. Additionally, as reported in other studies, vaginal stump suturing, which can be challenging with straight needle holders, can be performed from multiple angles, similar to robotic suturing (16). Conversely, articulating instruments are heavier than conventional ones, which can lead to physical strain on the surgeon's wrist, arm, or shoulder (26). To move the end-effector in the intended direction, coordinated movements of the wrist and arm are required, necessitating a learning curve even for experienced laparoscopic surgeons.

One of the key strengths of our study lies in its procedural consistency, as all surgeries were performed by two experienced, high-volume surgeons at a single institution, thereby minimizing variability related to surgical skill. Additionally, we have included surgical video documentation to illustrate the use of articulating instruments in hysterectomy, offering a valuable visual guide for surgeons seeking to adopt this technique. The structured description of surgical steps—particularly for colpotomy, avascular space creation, and suturing—adds further value by contributing to the standardization of laparoscopic technique using novel instruments. Notably, all 45 procedures were completed laparoscopically without conversion, supporting the clinical feasibility and safety of the approach. As one of the first prospective studies to explore this technology in gynecologic surgery, our findings may serve as a foundation for future comparative and multicenter investigations.

Despite its strengths, this study has several important limitations. First, the relatively modest sample size and the inclusion of patients with varied benign and malignant conditions limit the breadth of interpretation, especially with respect to oncologic outcomes or generalizability across disease subtypes. The cohort was not sufficiently powered to support subgroup analyses or statistically robust comparisons. Second, the observational nature of the study and lack of a control arm restrict the ability to draw causal inferences or directly compare the articulating instrument's performance with conventional or robotic systems. Third, while perioperative and some delayed complications were reported, the absence of extended follow-up precludes evaluation of long-term outcomes such as recurrence, functional recovery, and patient satisfaction. Lastly, although the cost-effectiveness of articulating instruments is a critical consideration in resource-conscious surgical settings, a formal economic assessment was not conducted and remains an important direction for future research.

In conclusion, the articulating laparoscopic instrument demonstrates potential as a practical and ergonomically favorable tool in laparoscopic hysterectomy, particularly for complex pelvic dissections. Nonetheless, our findings should be interpreted within the context of these limitations, and we strongly advocate for future comparative or randomized studies to more rigorously evaluate its clinical efficacy and broader applicability.

## Data Availability

The original contributions presented in the study are included in the article/Supplementary Material, further inquiries can be directed to the corresponding author.

## Supplementary material

Supplementary Video S1 Creation of an avascular space using an articulating instrument during dual-port laparoscopic hysterectomy.

Supplementary Video S2 Performing colpotomy using a monopolar articulating instrument during dual-port laparoscopic hysterectomy.

Supplementary Video S3 Vaginal cuff closure using a 2-0 absorbable barbed suture with an articulating needle holders.
