# Supplementary material for: Outcomes and surgical technique of laparoscopic hysterectomy using an articulating laparoscopic instrument: a prospective observational study
Source: Front Surg. 2025 Jun 24;12:1491767. doi: 10.3389/fsurg.2025.1491767 (PMC12234542; doi:10.3389/fsurg.2025.1491767)
Supplement: Supplementary file 4 [file Table1.docx]

Supplementary Material

**Supplementary Table S1.** Comparison of perioperative outcomes based on uterus weight

| Variable | Uterus ≥ 500 g  (n = 11) | Uterus < 500 g  (n = 34) | p-value |
| --- | --- | --- | --- |
| Weight of uterus (g) | 608.0 (512 – 875) | 135.5 (43 – 487) | < 0.001 |
| Age at operation (years) | 45.8 ± 5.0 | 48.2 ± 9.4 | 0.17 |
| BMI (kg/m^2^) | 24.4 (19.7–27.9) | 22.9 (19.2–41.9) | 0.92 |
| Prior abdominal surgeries | 6 (54.5) | 12 (35.2) | 0.25 |
| Main indication  Leiomyoma  Adenomyosis  Others | 8 (72.7)  3 (27.2)  0 (0.0) | 7 (20.5)  4 (11.8)  23 (67.6) | < 0.001 |
| Total operative time (min) | 84 (72–128) | 76 (44–156) | 0.26 |
| EBL (mL) | 100 (50–300) | 100 (50–200) | 0.11 |
| Hb decrease (g/dL) | 1.6 ± 0.9 | 1.5 ± 0.7 | 0.62 |
| Transfusion | 0 (0.0) | 1 (2.9) | 0.56 |
| Hospital stays (days) | 2 (2–2) | 2 (2–4) | 0.25 |
| Postoperative complication | 2 (18.1) | 4 (11.7) | 0.58 |

Data are presented as mean ± SD, median (range), or No. (%). SD, standard deviation; EBL, estimated blood loss; Hb = hemoglobin.
